# Giant Cell Urothelial Carcinoma of Bladder

**DOI:** 10.1155/2021/8021947

**Published:** 2021-07-15

**Authors:** Harshima Disvini Wijesinghe, Ajith Malalasekera

**Affiliations:** ^1^Department of Pathology, Faculty of Medicine, University of Colombo, Sri Lanka; ^2^Department of Anatomy, Faculty of Medicine, University of Colombo, Sri Lanka

## Abstract

Giant cell urothelial carcinoma is a rare variant of bladder cancer recognized by the current World Health Organization classification of urologic tumours. It is an aggressive tumour with a poor prognosis that usually presents at an advanced stage. It is characterized histologically by pleomorphic giant cells. We discuss a case of giant cell urothelial carcinoma presenting at an early stage in a previously well 62-year-old woman. Histology showed a tumour comprising pancytokeratin positive bizarre mononuclear and multi-nuclear giant cells admixed with areas of conventional urothelial carcinoma and carcinoma in situ. Three-month follow-up cystoscopy and magnetic resonance imaging showed no evidence of recurrence or pelvic lymphadenopathy.

## 1. Introduction

Giant cell urothelial carcinoma is a rare aggressive variant of urothelial carcinoma characterized by the presence of highly pleomorphic bizarre tumour giant cells. We report a case of giant cell urothelial carcinoma in a 62-year-old woman. The approach to diagnosis and differential diagnosis is discussed.

## 2. Case History

**Figure 1:**
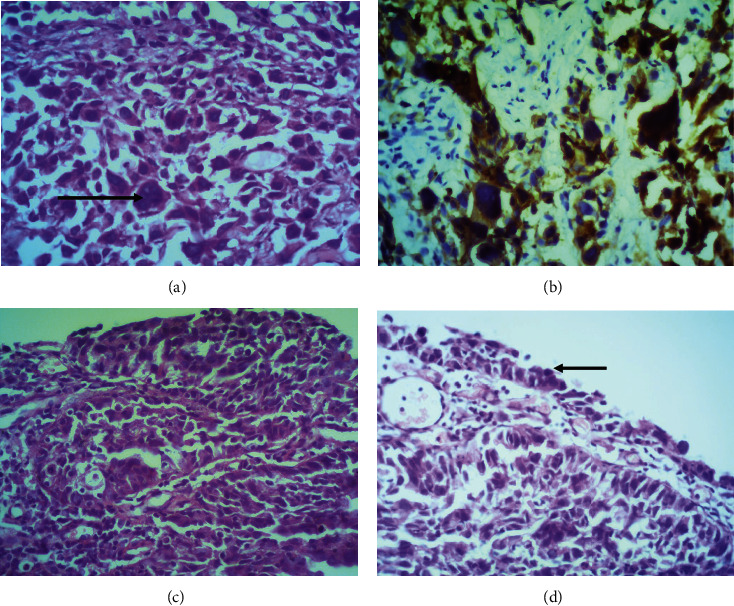
Histological appearance of the tumour. Giant cell urothelial carcinoma comprising sheets of pleomorphic cells with admixed bizarre mononuclear and multinuclear giant cells ((a), haematoxylin and eosin ×400) which were positive for pancytokeratin ((b), AE1/AE3 ×400) admixed with areas of conventional urothelial carcinoma (c) and carcinoma in situ (d).

A 62-year-old woman presented with visible haematuria. Cystoscopy showed a 15 mm papillary growth with a broad base in the left wall of the bladder. She underwent transurethral resection of the bladder tumour. The entire tumour was processed and examined microscopically. Microscopic examination showed bladder tissue infiltrated by a high-grade (Grade 3) urothelial carcinoma with a component of giant cell urothelial carcinoma. The tumour was composed predominantly of cells arranged in diffuse sheets and solid nests. The constituent cells had enlarged, markedly pleomorphic, hyperchromatic nuclei, and frequent mitoses. Admixed bizarre mononuclear and multinuclear giant cells were seen (Figure 1(a)). The giant cells were positive for pancytokeratin (AE1/AE3) (Figure 1(b)). Areas of conventional urothelial carcinoma (Figure 1(c)) with overlying carcinoma in situ were present (Figure 1(d)). The tumour showed extensive invasion of the lamina propria and muscularis mucosa, but the invasion of muscularis propria was not seen. Lymphovascular invasion was present. Follow up cystoscopy at three months did not show any recurrence in the bladder. There was no residual tumour or pelvic lymphadenopathy on a magnetic resonance imaging scan done at four months.

## 3. Discussion

Giant cell urothelial carcinoma is a rare aggressive carcinoma [1]. A variant of UC with highly pleomorphic tumour giant cells has been described in several publications since it was first reported in 1997 [2]. There are two case series and a few case reports that describe the clinical and pathological features of giant cell urothelial carcinoma [3–5] (Tables 1 and 2), including an interesting case of polyomavirus- (BK-) associated pleomorphic giant cell carcinoma of the urinary bladder associated with areas of trophoblastic differentiation [5].

Giant cell urothelial carcinoma has also been called pleomorphic giant cell carcinoma and large-cell undifferentiated carcinoma. Like most urothelial carcinomas, giant cell urothelial carcinoma occurs more commonly in older males (ages ranging from 53 to 92 years). It presents with haematuria, dysuria, or frequency [1.3.4]. It is characterized histologically by the presence of highly pleomorphic bizarre tumour giant cells similar to those seen in giant cell carcinoma of the lung. These tumours usually show muscularis propria invasion and extensive necrosis, although they were not identified in the present case. The current guidelines recommend that ideally all the bladder tumour tissue resected by transurethral resection is submitted, for pathological assessment. In large specimens where this is not feasible, it is recommended to submit the first 20 g (10 cassettes) of tissue plus one cassette for every additional 5 g [6]. This will facilitate accurate staging and histopathological subtyping of the tumour. This was a small tumour and it was sampled in its entirety.

The current World Health Organization (WHO) classification defines giant cell carcinoma as a rare form of infiltrating urothelial carcinoma resembling giant cell carcinoma of the lung in which the proliferating cells may appear undifferentiated [1]. Giant cell urothelial carcinoma shows expansile masses of the pleomorphic epithelioid tumour with bizarre anaplastic multinucleated and mononucleated tumour giant cells. Tumour cells have abundant cytoplasm and show frequent typical or atypical mitotic figures. Giant cell urothelial carcinoma has been reported in association with both conventional urothelial carcinoma and variants such as micropapillary, plasmacytoid, and lymphoepithelioma-like urothelial carcinoma [3–5]. It has also been reported with carcinomas with areas of trophoblastic differentiation [5]. The pleomorphic giant cell component, which accounted for 50% of the tumour in the present case, has been reported to range from 20% to 100% of the tumour [3].

Giant cell urothelial carcinoma must be distinguished from sarcomatoid carcinoma. The bladder and prostate cases with a significant spindle cell component [1] are called sarcomatoid carcinoma and are considered to be distinct from giant cell urothelial carcinoma which lacks a malignant spindle cell component. This is in contrast to organs such as the lung where according to the WHO classification of lung tumours, giant cell carcinoma is considered to be a subtype of sarcomatoid carcinoma [7].

Giant cell urothelial carcinoma must be differentiated from other primary bladder tumours that can contain giant cells, including osteoclast rich undifferentiated carcinoma and urothelial carcinoma with trophoblastic differentiation. The giant cells in giant cell urothelial carcinoma are morphologically different from the giant cells seen in these two entities [1, 8]. In contrast to giant cell urothelial carcinoma, osteoclast-rich undifferentiated carcinoma has a biphasic appearance. Giant cells resembling osteoclastic giant cells have numerous small bland nuclei and are present in a background of mononuclear cells [1, 8, 9]. These giant cells are positive for CD68 and LCA and negative for cytokeratins and epithelial membrane antigen.

Giant cell urothelial carcinoma must also be distinguished from metastasis from a giant cell carcinoma, melanoma, or sarcoma and direct extension from a pleomorphic giant cell carcinoma of the prostate. Coexisting carcinoma in situ and conventional urothelial carcinoma have been reported in the vast majority of cases [4] and were seen in this case. This helps to exclude a metastatic malignancy. It also raises the possibility that this is not a specific subtype of urothelial carcinoma but is instead a feature of extreme dedifferentiation. Small biopsies may not show areas of conventional urothelial carcinoma, carcinoma in situ, or variants of urothelial carcinoma making diagnosis difficult. In such cases, immunostaining, particularly with CK7, CK20, melanocytic markers, GATA3, and uroplakin III, needs to be performed.

The cells of the giant cell urothelial carcinoma component show positivity for CK 8/18 and AE1/AE3. Studies have also shown positivity for CK7, CK20, uroplakin III, and GATA3 in 90% of cases [4]. Some cases have shown positivity for p63 [4]. Uroplakin III has value as a marker for urothelial carcinoma. However, the use of this immunostain alone can be misleading, as carcinoma metastatic to the bladder can be positive. GATA3 is of greater utility [10]. When the differential diagnosis is giant cell urothelial carcinoma of the prostate, a panel including prostate-specific antigen, prostate-specific acid phosphatase, p63, and GATA3 would be diagnostic. Additionally, negativity for *β*HCG and CD68 helps to differentiate the giant cells from trophoblastic giant cells and osteoclast-type cells, respectively. Molecular characteristics of giant cell urothelial carcinoma are yet unknown [9].

This tumour has a very poor prognosis. Patients often have advanced stage cancer at presentation. Death within a short period, metastasis, or recurrent high-grade urothelial carcinoma has been reported in the majority. In the case series of 8 cases, reported by Lopez [3], all patients presented with ≥T3 disease. Six had lymph node metastases. Seven of the eight patients were either dead of disease or alive with metastases after fewer than two years' follow-up. In the case series reported by Samaratunga et al. [4], five of ten patients died within 12 months. One patient developed metastatic disease at 17 months following cystectomy, even though the initial staging on TURBT was pT1. Three other patients had recurrent high-grade urothelial carcinoma within three years. Only three of twenty-three patients reported in the literature were disease free for over three years. All three had pT3 disease and had undergone cystoprostatectomy, one following neoadjuvant chemotherapy [3, 4, 11].

This patient presented with T1 disease and was disease free at three-month follow-up. However, she had a stage T1, high-grade (Grade 3) tumour, and carcinoma in situ which places her in a high-risk category based on the European Association of Urology guidelines for the management of nonmuscle invasive bladder cancer. The probability of recurrence and progression at one year is 24% and 17%, respectively [12]. Based on the above guidelines, she would require follow-up with cystoscopy and cytology every three months for a period of two years, every six months thereafter until five years and then yearly. Annual upper tract imaging with computed tomography-intravenous urography (CT-IVU) or IVU is also recommended [12].

## Figures and Tables

**Table 1:** Clinical features of reported cases of giant cell urothelial carcinoma.

Case series/case report	Number of cases	M : F	Age (years)	Presentation	Type of specimen	Prognosis
Lopez et al. 2009 [3]	8	3 : 1	Mean age—67 Range (55-88)	Haematuria—8 (100%) Frequency—2 (25%) Dysuria—2 (25%)	Transurethral resection of bladder—3 (37.5%) Cystoprostatectomy—3 (37.5%) Cystectomy with hysterectomy—2 (25%)	Death within 17 months—5 (62.5%) Metastasis at 11 and 19 months—2 (25%) Disease free at 74 months—1 (12.5%)

Samaratunga et al. 2016 [4]	13 (IHC and follow-up available in 10 cases)	9 : 4	Mean age—72 Range—53-92	Haematuria—13 (100%) Voiding dysfunction—7 (53.8%)	Cystoprostatectomy—2 (15.4%) Transurethral resection specimens of bladder (TURBT) —11 (84.6%)	Death within 12 months—5 (50%) Metastasis at 17 months—1 (10%) Recurrent high grade tumour—3 (30%) Disease free at 46 months following cystoprostatectomy—1 (10%)

Alexiev et al. 2013 [5]	1	Male	77	Renal transplant recipient—12 years post-transplant voided urine samples were positive for malignancy and decoy cells	Cystoprostatectomy	Distant metastasis at 4 months

Kimura et al. 2019 [11]	1	Male	73	Haematuria	Transurethral resection of bladder tumour followed by radical cystectomy and lymphadenectomy following two cycles of neoadjuvant chemotherapy with gemcitabine and cisplatin.	Disease free at 4 years

**Table 2:** Pathological features of reported cases of giant cell urothelial carcinoma.

Case series/case report	CIS/conventional urothelial CA/variants of UC	Proportion of giant cell UC	IHC	Stage at presentation
Lopez et al. 2009 [3]	Lymphoepithelioma like urothelial CA—1 (12.5%) Micropapillary carcinoma—1 (12.5%)	20-100% 100%—1 (12.5%)	CK8/18—8 (100%) AE1/AE3—8 (100%) EMA—8 (100%) CK7—8 (100%)	pT3—6 (75%) pT4—2 (25%) Lymph node metastasis—6 (75%)

Samaratunga et al. 2016 [4]	CIS—5 (38.5%) Conventional UC—8 (61.5%) Micropapillary UC—3 (23.15) Plasmacytoid UC—1 (7.7%) None—3 (23.1%)	40-100% 40%—3 (23.1%) 50%—3 (23.1%) 80%—3 (23.1%) 95%—1 (7.75) 100%—3 (23.1%)	CK8/18—10 (100%) AE1/AE3—10 (100%) CK7—9 (90%) CK20—9 (90%) P63—3 (30%) Uroplakin—9 (90%) GATA 3—9 (90%)	pT1—8 (61.5%) pT2—3 (23.15%) pT3—2 (15.4%)

Alexiev et al. 2013 [5]	Conventional high-grade urothelial carcinoma (20%) Trophoblastic differentiation (10%).	70%	Pleomorphic giant cells were positive for CK903, CK7 p63, p53, and p16. HCG expression was present in scattered giant cells. Tissue sections from the primary pleomorphic giant cell carcinoma and the omental metastasis were positive for SV40.	pT3bNxM1 (omental node metastasis were present)

Kimura et al. 2019 [11]	Conventional urothelial carcinoma	70%	Not available	cT3bN2M0→ypT3aN0M0

## Data Availability

No data were used.

## References

[1] Moch H., Humphrey P. A., Ulbright T. M., Reuter V. (2016). *WHO Classification of Tumours of the Urinary System and Male Genital Organs*.

[2] Eble J. N., Young R. H. (1997). Carcinoma of the urinary bladder: a review of its diverse morphology. *Seminars in Diagnostic Pathology*.

[3] Lopez-Beltran A., Blanca A., Montironi R., Cheng L., Regueiro J. C. (2009). Pleomorphic giant cell carcinoma of the urinary bladder. *Human Pathology*.

[4] Samaratunga H., Delahunt B., Egevad L. (2016). Pleomorphic giant cell carcinoma of the urinary bladder: an extreme form of tumour dedifferentiation. *Histopathology*.

[5] Alexiev B. A., Papadimitriou J. C., Chai T. C., Ramos E., Staats P. N., Drachenberg C. B. (2013). Polyomavirus (BK)-associated pleomorphic giant cell carcinoma of the urinary bladder: a case report. *Pathology Research and Practice*.

[6] Shanks J. H., Chandra A., McWilliam L., Varma M. (2013). *Dataset for tumours of the urinary collecting system (renal pelvis, ureter, urinary bladder and urethra)*.

[7] Travis W. D., Brambilla E., Burke A. P., Marx A., Nicholson A. G. (2015). *WHO Classification of Tumours of the Lung, Pleura, Thymus and Heart*.

[8] Baydar D., Amin M. B., Epstein J. I. (2006). Osteoclast-rich undifferentiated carcinomas of the urinary tract. *Modern Pathology*.

[9] Lopez-Beltran A., Henriques V., Montironi R., Cimadamore A., Raspollini M. R., Cheng L. (2019). Variants and new entities of bladder cancer. *Histopathology*.

[10] Klopfer K., Delahunt B., Adamson M., Samaratunga H. (2014). The value of uroplakin III in distinguishing variants of primary bladder urothelial carcinoma from malignancy metastatic to the urinary bladder. *Anticancer Research*.

[11] Kimura H., Uemura Y., Megumi Y., Tachibana M., Fukuzawa S. (2019). A case Of cT3bN2M0 pleomorphic giant cell carcinoma of the bladder without recurrence after neoadjuvant chemotherapy and radical cystectomy for 4 Years. *Hinyokika Kiyo*.

[12] Babjuk M., Burger M., Compérat E. M. (2019). European Association of Urology Guidelines on Non-muscle-invasive Bladder Cancer (TaT1 and Carcinoma In Situ) - 2019 Update. *European Urology*.

